# RIP1 Mediates Manzamine-A-Induced Secretory Autophagy in Breast Cancer

**DOI:** 10.3390/md21030151

**Published:** 2023-02-25

**Authors:** Xuan Wang, Yuanpeng Liu, Huan Qin, Guocui Qi, Xuehong Chen, Yi Lyu, Yantao Han

**Affiliations:** 1 Department of Pharmacology, School of Basic Medicine, Qingdao University, Qingdao 266071, China; 2 Department of Biology, Hobart and William Smith Colleges, Geneva, NY 14456, USA

**Keywords:** manzamine A, secretory autophagy, RIP1, sEVs, breast cancer

## Abstract

Cancer-derived small extracellular vesicles (sEVs) serve as critical mediators of cell-to-cell communication. Manzamine A (MA), a unique marine-derived alkaloid with various bioactivities, exerts anticancer effects against several kinds of tumors, but it remains unclear whether it has the same activity against breast cancer. Here, we proved that MA inhibits MDA-MB-231 and MCF-7 cell proliferation, migration, and invasion in a time- and dose-dependent manner. In addition, MA promotes autophagosome formation but suppresses autophagosome degradation in breast cancer cells. Importantly, we also found that MA stimulates sEVs secretion and increases autophagy-related protein accumulation in secreted sEVs, further potentiated by autophagy inhibitor chloroquine (CQ). Mechanistically, MA decreases the expression level of RIP1, the key upstream regulator of the autophagic pathway, and reduces the acidity of lysosome. Overexpression of RIP1 activated AKT/mTOR signaling, thus attenuating MA-induced autophagy and the corresponding secretion of autophagy-associated sEVs. Collectively, these data suggested that MA is a potential inhibitor of autophagy by preventing autophagosome turnover, and RIP1 mediates MA-induced secretory autophagy, which may be efficacious for breast cancer treatment.

## 1. Introduction

Marine invertebrates have been considered as a vital source of pharmacologically bioactive natural products [1]. The manzamines are a class of β-carboline alkaloids extracted from marine sponges with a nitrogen-containing polycyclic system [2]. As a representative compound of this class, Manzamine A (MA) has been reported to have anti-malarial, anti-microbial, anti-inflammatory, anti-aging, anti-atherosclerosis activities [3,4,5,6], and so on. Recently, a growing body of evidence demonstrates that it also has anticancer effects on several types of cancer, such as cervical cancer [7], pancreatic cancer [8], colorectal cancer [9], and glioblastoma [10], by the induction of cell apoptosis, cell autophagy, cell cycle arrest, and the inhibition of cell migration/invasion. However, whether it has this activity against breast cancer and the underlying mechanism of it remain unclear. 

Small extracellular vesicles (sEVs) are nanosized membrane vesicles with diameters from 50 to 150 nm that are extracellularly secreted after the fusion of multivesicular bodies (MVBs) (formed by endocytic vesicles) with plasma membrane [11]. As an important member of EVs, sEVs play pivotal roles in distinct biological functions due to containing proteins, lipids, and nucleic acids that are derived from parent cells [12], including the contribution to the progression of metabolic and cardiovascular diseases, neurodegenerative diseases, as well as the development of cancers via intercellular communication in cancer cells, and so on [13,14,15]. Currently, increasing studies have shown that secretory autophagy intersects with the biogenesis and secretion of sEVs [16,17]. Namely, beyond the consolidated role in degrading and recycling cellular waste, the autophagic-lysosomal system is closely correlated to exocytosis of sEVs. Lysosomes are acidic organelles filled with a variety of hydrolytic enzymes that degrade intracellular components from autophagy and extracellular substances from endocytosis [18]. Therefore, autophagosomes will fuse with MVBs to release sEVs when lysosomal dysfunction and lysosomal acidity are bound to affect the content and secretion of sEVs [19]. In our study, we found that MA increases autophagosome accumulation but reduces autophagosome degradation via decreasing lysosomal acidity. Meanwhile, MA promotes the release of sEVs rich in autophagy-related proteins. 

Receptor interacting protein 1 (RIP1) is a threonine/serine protein kinase with multiple functions, associated with the regulation of cell autophagy, apoptosis, necroptosis, proliferation, mitochondrial metabolism, and cytokine/chemokine production by phosphorylating the critical mediators of the corresponding signal transduction pathways [20,21]. Hence, we postulated that RIP1 may have influences on MA-induced secretory autophagy in breast cancer.

## 2. Results

### 2.1. MA Inhibits Breast Cancer Cell Proliferation

**Figure 1:**
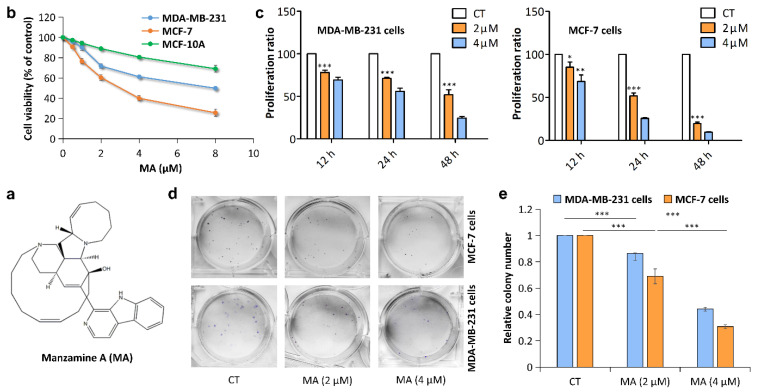
MA reduces cell proliferation in breast cancer cells. (**a**) Chemical structure of MA. (**b**) MTT assay was conducted to measure cell viability of MCF-10A cells, MDA-MB-231 cells, and MCF-7 cells treated with MA at different concentrations (0, 0.5, 1, 2, 4, and 8 μM) for 24 h. (**c**) CCK-8 assay was used to detect cell proliferation in MDA-MB-231 cells and MCF-7 cells incubated with MA at different concentrations (0, 2, 4 μM) for 12 h, 24 h, 48 h, respectively. Cell proliferation ratio was expressed as percentage of absorbance from MA group compared to CT group. (**d**) Colony formation assay was performed to evaluate the long-term effects of MA treatment with different concentrations (0, 2, 4 μM) on the growth of MDA-MB-231 and MCF-7 cells. Clonogenic images were photographed on day 14. (**e**) Relative colony numbers of the cells were calculated. All data were presented as the mean ± S.D. for three independent experiments. * *p* < 0.05; ** *p* < 0.01, *** *p* < 0.001.

To explore the effects of MA (Figure 1a) on breast cancer cell proliferation, we performed the MTT assay on MCF-10A, MDA-MB-231, and MCF-7 cells at concentrations of 0, 0.5, 1, 2, 4, and 8 μM for 24 h. The results displayed that MA had cytotoxic effects on MDA-MB-231 and MCF-7 cells but was non-toxic to normal breast epithelial MCF-10A cells in a dose-dependent manner (Figure 1b). The IC50 values of MA in MDA-MB-231 and MCF-7 cells are shown in Table 1 compared with those of paclitaxel (PTX). Additionally, we determined the proliferation ratio of MDA-MB-231 and MCF-7 cells treated with MA (2, 4 μM) for different times (12, 24, and 48 h) by using CCK-8 assay, and we found that MA significantly inhibited breast cancer cell proliferation in a time- and dose-dependent manner compared to the CT (control) group (Figure 1c). The antiproliferation effect was further supported by colony formation assay. The 24 h MA (0, 2, 4 μM)-treated cells were seeded in 6-well plates and then cultured for 2 weeks. We observed that MA dose-dependently decreased the colony numbers in both MCF-7 and MDA-MB-231 cells after exposure to MA (Figure 1d–e), indicating a long-term ability of MA to inhibit breast cancer cell growth.

### 2.2. MA Inhibits Breast Cancer Cell Migration and Invasion

**Figure 2:**
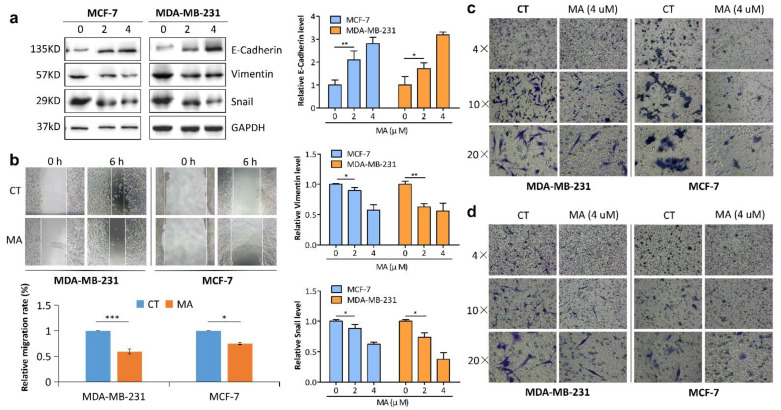
Anti-EMT properties of MA. (**a**) Western blot analysis of EMT-related proteins in MDA-MB-231 cells and MCF-7 cells grown in the absence or presence of MA for 24 h. (**b**) Wound healing assay was carried out to evaluate the migration abilities of MDA-MB-231 cells and MCF-7 cells. Representative images showing migrating cells after treatment with MA (0, 2 μM) for 6 h. (**c**,**d**), Transwell migration and invasion assays of MDA-MB-231 cells and MCF-7 cells following treatment with 4 μM MA or DMSO for 24 h. Photomicrograph of migrated cells and invaded cells were viewed at different magnification (4×, 10×, 20×). All data were presented as the mean ± S.D. for three independent experiments. * *p* < 0.05; ** *p* < 0.01.

To investigate whether MA affected cell migration and invasion, we detected the Epithelial–Mesenchymal Transition (EMT)-associated protein markers in MA-treated MDA-MB-231 and MCF-7 cells by Western blotting. The results displayed that MA dose-dependently decreased the proteins level of Snail and Vimentin (mesenchymal markers), while it increased the protein level of E-cadherin (epithelial marker) (Figure 2a). Additionally, we assessed the effect of MA on the migration of breast cancer cells using scratch wound assay. The result showed that MA inhibited the relative migration of the cells at 6 h after wounding versus the CT group (Figure 2b). Consistent with the result by scratch wound assay, the images of transwell assay further showed that MA significantly reduced the number of migrated cells and invaded cells compared to the controls photographed by a microscope at magnification of 4×, 10×, and 20× (Figure 2c,d). Altogether, these results demonstrated that MA inhibits the migration and invasion in breast cancer cells. 

### 2.3. MA Promotes Autophagosome Formation and Inhibits Autophagosome Degradation in Breast Cancer Cells

**Figure 3:**
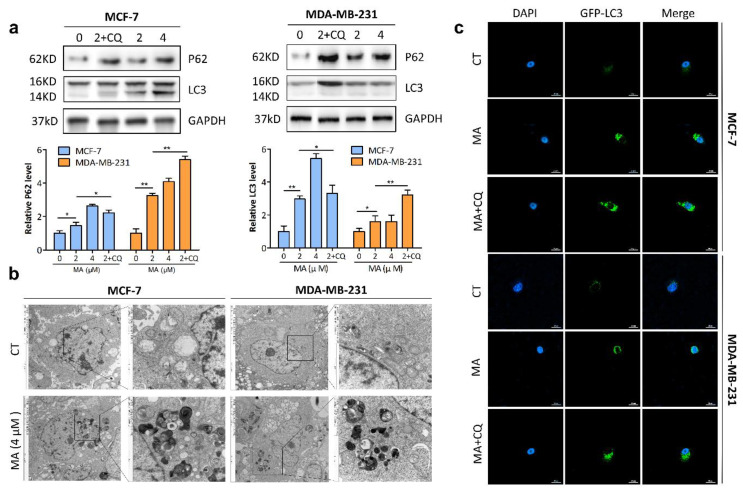
MA facilitates autophagosome formation and inhibits autophagosome degradation in breast cancer cells. (**a**) Immunoblot analysis was preformed to detect autophagy-related proteins in MDA-MB-231 cells and MCF-7 cells treated with indicated concentrations of MA (0, 2, 4 μm/L) and/or CQ (20 μm/L) for 24 h. All the data were expressed as the mean ± S.D. of triplicate experiments. * *p* < 0.05; ** *p* < 0.01. (**b**) TEM was conducted to observe the autophagosomes in MDA-MB-231 cells and MCF-7 cells after treatment with MA (4 μm/L) for 24 h. (**c**) MDA-MB-231 cells and MCF-7 cells transfected with GFP-LC3 were treated with or without 4 μm MA and/or 20 μm CQ for 24 h and images were obtained by using confocal microscopy. Scale bar: 20 μm.

To explore the effect of MA on autophagy in breast cancer cells, MDA-MB-231 and MCF-7 cells were treated with MA for 24 h followed by Western blot analysis. As shown in Figure 3a, the accumulation of autophagosome marker LC3Ⅱ was dose-dependently increased. However, P62, another marker that inversely correlates with autophagic activity [23], was also remarkably increased in a dose-dependent manner, suggesting the induction of autophagosome formation but the inhibition of autophagosome degradation after MA treatment. Furthermore, the above regulatory effects of MA on autophagy can be enhanced by CQ (inhibitor of autophagosome–lysosome fusion). In addition, TEM, the gold standard for observing the morphology of autophagosomes, was conducted. As anticipated, double- or multi-membrane structures were accumulated in MA-treated MCF-7 and MDA-MB-231 cells (Figure 3b). MCF-7 and MDA-MB-231 cells transiently expressing GFP-LC3 incubated with MA and/or CQ for 24 h were imaged by confocal microscopy and the results displayed distinct punctae distribution in line with autophagosome formation. Accordingly, breast cancer cells treated with MA in combination with CQ resulted in an significant increase in fluorescence intensity compared to the cells treated with MA alone (Figure 3c). Overall, these data suggested that MA inhibits the last step of the autophagic process by preventing autophagosome turnover. 

### 2.4. RIP1 Mediates MA-Induced Autophagy through Akt/mTOR Pathway

**Figure 4:**
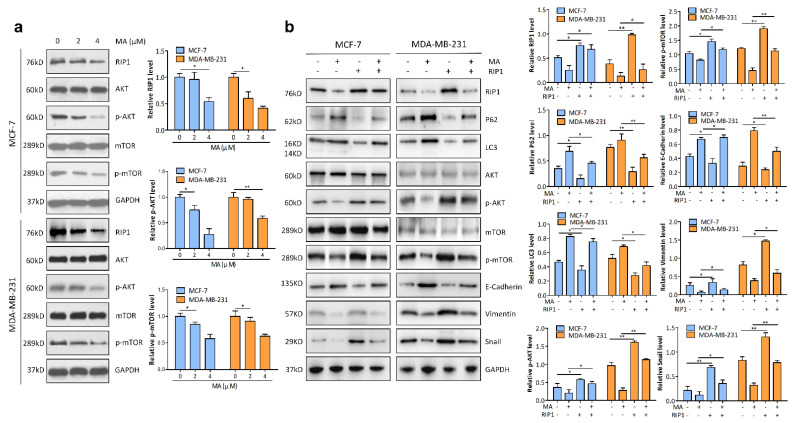
RIP1 mediate MAinduced autophagy through AKT/mTOR pathway. (**a**) Immunoblot analysis was performed with antibodies as indicated in MCF-7 and MDA-MB-231 cells treated with increased concentrations (0, 2, 4 μM) of MA. (**b**) Immunoblot analysis was performed to detect the phosphorylation status of AKT and mTOR and the expression levels of autophagy-related proteins (LC3 and P62) and EMT-related proteins (E-Cadherin, Vimentin, and Snail) in MCF-7 cells and MDA-MB-231 cells transfected with/without RIP1 in the presence or absence of MA as indicated. All data are presented for three independent experiments. * *p* < 0.05; ** *p* < 0.01.

The AKT/mTOR axis is a classic signaling pathway in cancers that blocks catabolic activities, such as autophagy [24]. We therefore examined whether the AKT/mTOR pathway is required for MA-induced autophagy. After being incubated with increasing concentrations (0, 2, 4 μm) of MA for 24 h, MCF-7 and MDA-MB-231 cells were harvested for immunoblotting assay. The results showed that the phosphorylation level of AKT and mTOR were dose-dependently decreased after MA treatment. We also detected the expression level of RIP1 kinase, a key upstream regulator that controls autophagy by phosphorylating the critical components of the signaling pathways [25]. The results showed that the expression of RIP1 was dose-dependently down-regulated in MCF-7 and MDA-MB-231 cells treated with MA (Figure 4a). Moreover, overexpression of RIP1 significantly increased phosphorylation levels of AKT and mTOR, reduced the accumulation of LC3II and P62, and greatly promoted EMT ( E-Cadherin↓, Snail, and Vimentin↑) compared with the non-transfection group (Figure 4b), suggesting that RIP1 mediates MA-induced autophagy through the AKT/mTOR pathway in MCF-7 and MDA-MB-231 cells. 

### 2.5. MA Reduces Lysosomal Acidity in Breast Cancer Cells 

**Figure 5:**
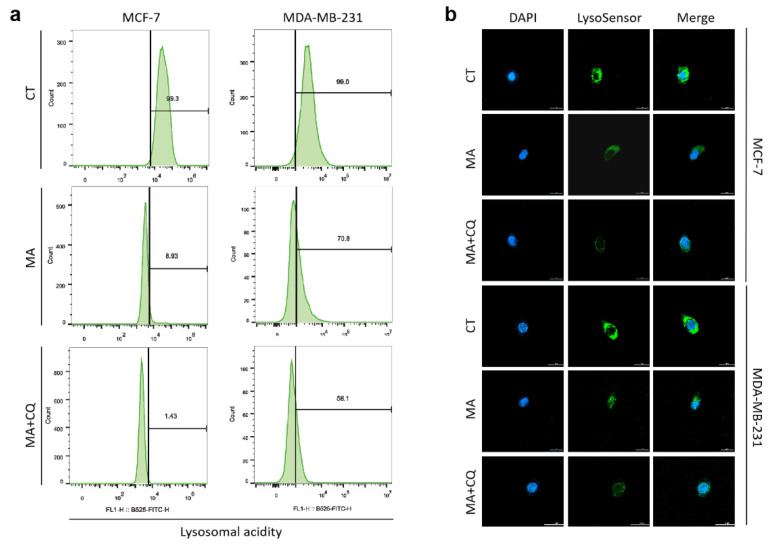
MA reduces lysosomal acidity in breast cancer cells. (**a**) Flow cytometry was performed to detect lysosomal acidity by LysoSensor™ Green DND-189 in MDA-MB-231 cells and MCF-7 cells treated with MA (4 μM) or/and CQ (20 μM) for 24 h. Numbers in histograms indicate percentage (%) of cells with decreased fluorescence intensity compared to cells treated with DMSO. (**b**) Immunofluorescence microscopy was used to analyze lysosomal acidity by Lysosensor™ green pH indicator in MDA-MB-231 cells and MCF-7 cells with 4 μM MA and/or 20 μM CQ treatment for 2 h at a magnification of 60×.

The low intralysosomal pH activates resident hydrolases responsible for the degradation of various cargos delivered by autophagic processes [26]. To determine whether the reduction in the degradation of autophagosomes produced by MA was correlated with lysosomal acidity, MCF-7 and MDA-MB-231 cells were treated with MA and/or CQ for 24 h followed by the acidic probe lysosensor dye staining, and then analyzed by flow cytometry and visualized by immunofluorescence microscopy, separately. The results of the flow cytometry analysis demonstrated that MA treatment resulted in a marked decrease in intralysosomal acidity when compared to the CT group, and CQ could further enhance this effect (Figure 5a). Consistent with the result by flow cytometry analysis, immunofluorescence showed a significant decrease in fluorescent intensity in MA-treated cells compared to DMSO-treated cells, which was further attenuated by CQ treatment (Figure 5b). These data indicated that MA reduces lysosomal acidity in breast cancer cells, which might be the reason why the degradation of autophagosomes is impaired by MA.

### 2.6. The Characterization of sEVs

**Figure 6:**
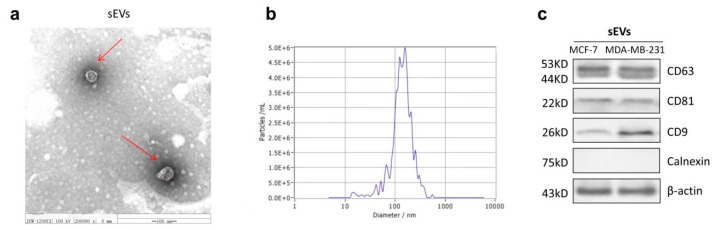
Characterization of sEVs. (**a**) Morphology of sEVs isolated from MDA-MB-231 cells under TEM, scale bar represents 100 nm. (**b**) Size distribution of sEVs derived from MDA-MB-231 cells using NTA. (**c**) Western blot analysis of sEVs protein markers in MDA-MB-231 cells and MCF-7 cells.

TEM was used to confirm the morphology of sEVs. We observed the characteristic rounded and typical bilayer membrane vesicles with heterogeneous size from purified sEVs released by MDA-MB-231 cells (Figure 6a). Next, we characterized sEVs to determine the size distribution by nanoparticle tracking analysis (NTA). The result showed the size was approximately between 50 and 150 nm (Figure 6b). In addition, Western blot analysis showed that sEVs expressed the sEV-specific markers (CD63, CD81, and CD9) and did not express the negative Calnexin marker (Figure 6c). These results are in line with the current recommendations of the International Society for Extracellular Vesicles (MISEV).

### 2.7. RIP1 Mediates MA-Induced Secretory Autophagy

**Figure 7:**
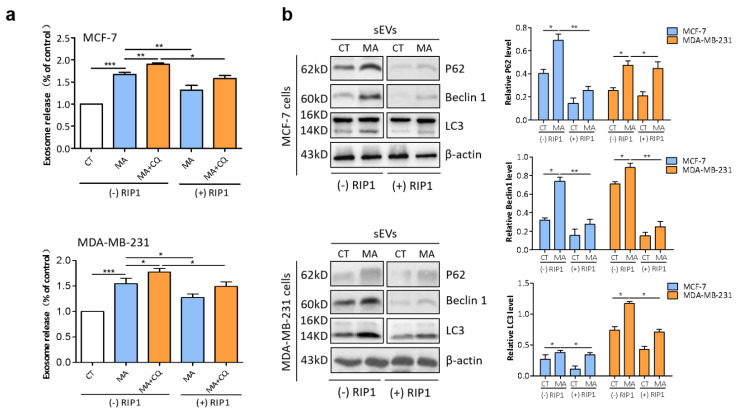
MA induces the secretion of autophagosome-derived sEVs. (**a**) AchE assay was conducted to determine sEVs release of MDA-MB-231 and MCF-7 cells treated with 4 μM MA and/or 20 μM CQ for 24 h. (**b**) Western blot analysis of autophagy-related proteins in sEVs extracted from MDA-MB-231 and MCF-7 cells treated with 4 μM MA for 24 h. The data were expressed as the mean ± S.D. of triplicate experiments. * *p* < 0.05, ** *p* < 0.01, *** *p* < 0.001.

Increasing evidence indicates that autophagosomes fused with MVBs [27,28]. To investigate whether MA affected secretion of SEVs, we performed an AChE assay to determine the release of sEVs. The results showed that the quantity was prominently increased in sEVs secreted by MDA-MB-231 and MCF-7 cells after MA treatment in comparison with the sEVs derived from DMSO-treated cells; moreover, CQ could further potentiate this effect, while RIP1 overexpression could attenuate this effect (Figure 7a). Subsequently, to clarify whether MA affected the composition of sEVs, immunoblot analysis was conducted to examine the expression levels of autophagy-associated proteins. The data showed that the expression levels of P62, LC3II, and beclin1 were prominently increased in sEVs released from MDA-MB-231 and MCF-7 cells after MA treatment compared with the sEVs purified from DMSO-treated cells. However, RIP1 overexpression could attenuate this effect (Figure 7b). Altogether, these findings indicated that MA induces secretory autophagy in breast cancer cells. 

## 3. Discussion

Natural products remain an unrivaled source of drug leads covering unique chemical space and providing significant therapeutic value for the control of cancers resistant to current drugs [7]. In the present study, we confirmed that a marine-derived natural product, MA, time- and dose-dependently suppresses breast cancer cell proliferation by MTT assay and colony formation assay. In addition, we also detected the expression levels of the proteins involved in EMT, a key factor contributing to tumor migration and invasion [29], and the results showed that the expression of epithelial cell marker E-Cadherin was up-regulated, while mesenchymal markers Vimentin and Snail were down-regulated in MCF-7 and MDA-MB-231 cells after exposure to MA as compared with the CT group, indicating that MA inhibits the metastasis of breast cancer cells, and the same results were also obtained by scratch wound assay and transwell assays. 

Then, Western blot analysis was conducted to determine whether MA affects autophagy in breast cancer cells, and the findings showed that MA dose-dependently elevated the levels of autophagic substrate p62/SQSTM1 and autophagosome marker LC3-II. Under physiological conditions, p62 protein was barely detectable due to rapid degradation in the lysosomal compartment during the autophagy process [30]. Meanwhile, the elevation of LC3-II can be due to enhanced autophagy initiation or reduced degradation in the lysosome [31]. Taken together, the highly enriched p62 and LC3-II implicate the accumulation of autophagosomes and the impairment of degradative autophagy [32]. Subsequently, further research for monitoring autophagosomes by using fluorescent-tagged LC3 plasmids (GFP-LC3 ) as well as TEM were conducted and we found that MA stimulates the formation of autophagosomes. Additionally, the above effects could be facilitated by CQ. Overall, these data revealed the block of autophagosome-lysosome fusion and the prevention of autophagosome turnover. The AKT/mTOR pathway is one of the major regulators of autophagy [33], we therefore examined whether the AKT/mTOR pathway is required for MA-induced autophagy. Our study showed that MA down-regulated the phosphorylation level of AKT and mTOR in a dose-dependent manner. Moreover, the expression level of RIP1 kinase was significantly decreased in MA-treated breast cancer cells. However, the overexpression of RIP1 increased the phosphorylation level of AKT and mTOR and decreased the accumulation of LC3II and P62 in MCF-7 and MDA-MB-231 cells after MA treatment, suggesting RIP1 mediates MA-induced autophagy through the AKT/mTOR pathway.

The low intralysosomal pH stimulates resident hydrolases responsible for the degradation of various cargos delivered by autophagic processes [26]. Therefore, the Lysosensor™ Green DND-189 reagents were used for indicating changes in the lysosomal concentration of H+. We found that MA reduces lysosomal acidity by using flow cytometric analysis and immunofluorescence microscopy in breast cancer cells. Meanwhile, CQ, blocking autophagic flux by impairing autophagosome-lysosome fusion [34], in combination with MA, could further reduce lysosomal acidity in breast cancer cells. 

To clarify whether autophagosomes fuse with MVBs after the inhibition of lysosomes, the release of sEVs were detected by AchE assay following the identification of sEVs in terms of shape, size distribution, and specific protein markers. We found that MA stimulates sEV release in comparison with the CT group. Additionally, this effect was further potentiated by CQ, while it was attenuated by RIP1 overexpression in MDA-MB-231 and MCF-7 cells. Importantly, our results displayed the accumulation of autophagy-associated proteins (P62, Beclin 1, and LC3) in sEVs secreted by breast cancer cells treated with MA, but they were attenuated in sEVs derived from RIP1-transfected MDA-MB-231 and MCF-7 cells, indicating that autophagosomes can fuse with either lysosomes or MVBs, and further suggesting that the release of sEVs may compensate for lysosomal dysfunction. An influence of lysosome function on EV biogenesis suggests a mechanism for the coordinated regulation of autophagy and EV release under physiological conditions. In conclusion, we demonstrated the anticancer effects of MA with considerable promise in targeting breast cancer cells. 

## 4. Materials and Methods

### 4.1. Cell Lines and Reagents 

Normal breast epithelial cell line MCF10A and human breast cancer cell lines MCF-7 and MDA-MB-231 were obtained from American Type Culture Collection (ATCC), and separately maintained in Dulbecco’s Modified Eagle’s Medium and RPMI 1640 medium supplemented with 10% fetal bovine serum (FBS) at 37 °C in a humidified incubator containing 5% CO_2_. Cells were passaged three times a week. The following reagents were commercially obtained: MA (purity = 99.29%) and CQ were from MedChemExpress (MCE). MA was dissolved in DMSO at a concentration of 5 mM by means of ultrasonic and the final dissolution was clear. Lipofectamine 2000 Reagent was from Invitrogen. LysoSensor™ Green DND-189 was from YEASEN. Antibodies against RIP1, SQSTM1/p62, LC3, GAPDH, and Goat anti-rabbit secondary antibody were from Elabscience (Houston, TA, USA); antibodies against Vimentin, CD63, CD9, and Calnexin were from Abcam (MA, USA); the antibody against E-cadherin was from BD Biosciences (Franklin Lakes, NJ, USA); the antibodies against phospho(Ser473)-AKT (p-AKT), AKT, phospho-mTOR (p-mTOR), mTOR, Snail, Beclin1, and CD81 were from Cell Signaling Technology (Danvers, MA, USA).

### 4.2. Cell Viability Assay

Cell viability was detected by MTT. Briefly, MDA-MB-231 cells, MCF-7 cells, and MCF10A cells were seeded into 96-well plates and cultured for 24 h. Then, the cells were treated with different concentrations of MA (0, 0.5, 1, 2, 4, 8 µM) for another 24 h. Subsequently, 20 µl MTT was added to each well for 4 h. Absorbance was measured using a microplate reader (Molecular Devices, Sunnyvale, U.S.) at 450 nm. Cell viability was calculated as the absorbance of the treated cells relative to that of untreated cells and presented as the mean ± SD of three independent experiments. IC50 values were calculated according to the log-probit equation (y = 0.4006x + 0.1411, R2 = 0.9823; y = 0.5532x + 0.2474, R2 = 0.9965) obtained via log-probit concentration–response effects of MA in MCF7 and MDA-MB-231 cells, and the results were presented as the mean ± SD. 

### 4.3. Cell Proliferation Assay

Cell proliferation was measured by the cell counting method using Cell Counting Kit-8 (CCK-8, Dojindo Laboratories), according to the manufacturer’s protocol. Briefly, MDA-MB-231 cells and MCF-7 cells were seeded in 96-well flat-bottom plates and cultured for 24 h prior to MA treatment with different concentrations (0, 2, 4 µM) for 12 h, 24 h, and 48 h, separately, then 10 μL CCK-8 was added to each well in the plate, followed by incubation for 1 h. The absorbance was read at an excitation of 450 nm and an emission of 590 nm using a microplate reader. All experiments were repeated 3 times in duplicates, and results were shown as means from three independent experiments.

### 4.4. Colony Formation Assay

A clonogenic assay was performed as described. Briefly, breast cancer cells were treated with MA (0, 2, 4 µM) for 24 h. Then, cells were trypsinized and dispensed into individual wells of a 6-well plate in triplicate at densities of 300 cells per well. After 14 days of incubation at 37 °C, the cells were fixed and stained with 0.25% crystal violet to visualize colonies, and colonies were counted manually.

### 4.5. Scratch Wound Assay

Scratch wound assay was performed to evaluate cell migration following our previous publication [35]. Briefly, MDA-MB-231 cells and MCF-7 cells were plated into 6-well plates and grown to monolayers, then the monolayers were scratched with sterile 200 µL pipette tips, washed with PBS 3 times, and subsequently cultured in serum-free culture medium containing MA (0, 2 μM). Photos were captured at different times (0, 6 hours) after wounding under an inverted microscope (Leica microsystems, Wetzlar, Germany). The scratch gap distance was quantitatively determined by ImageJ software (National Institutes of Health). The closure area of wound was calculated as follows: migration area (%) = (A_1_ − A_2_)/A1 × 100, where A_1_ represents the area of initial wound area, A_2_ represents the remaining area of wound at the metering point.

### 4.6. Transwell Assay

Transwell chambers coated with or without Matrigel (BD bioscience, SanJose, CA, USA) were used to determine cell migration and invasion. For the cell migration, breast cancer cells untreated or treated with MA for 48 h were seeded into the upper chambers in serum-free medium, while complete medium was added into the lower chambers. After incubation at 37 °C with 5% CO_2_ for 24 h, The upper chamber was cleaned with a cotton swab and the lower chamber was fixed with 4% paraformaldehyde, stained with 0.1% crystal violet, and then imaged by an inverted microscope (Zeiss, Oberkochen, Germany). The procedures for the cell invasion were the same as for the cell migration except that the upper compartment was precoated with 100 μL of Matrigel. 

### 4.7. Western Blot Analysis

Western blot was used to analyze protein expression as described previously [36]. In brief, total proteins of cells or sEVs were extracted with RIPA buffer (Elabscience) and determined by BCA assay according to the instructions. Equal proteins were loaded on SDS-PAGE gels and transferred to nitrocellulose membranes, then the membranes were blocked with 5% fat-free milk and subsequently incubated with primary antibodies at 4 ℃ overnight. After incubation with peroxidase-conjugated secondary antibodies, target bands were visualized under Chemiluminescence Imaging System (Bio-Rad, Hercules, CA, USA) and the expression levels of the target proteins were analyzed by ImageJ Software (Bio-Rad, USA).

### 4.8. Transmission Electron Microscopy (TEM)

TEM was conducted as described previously [37]. Briefly, breast cancer cells treated with MA were collected and fixed in 4% glutaraldehyde overnight, and post-fixed with 1% osmium tetroxide for 4 h. The sample was embedded after dehydration in ethanol and infiltration with propylene oxide. Sections (50 nm) were prepared and double stained with uranyl acetate and lead citrate, and then examined by transmission electron microscopy (HITACHI HT7700, Tokyo, Japan).

### 4.9. Confocal Microscropy

Autophagosomes formation was evaluated by GFP-LC3 vectors transfected into breast cancer cells using Lipofectamine 2000 Reagent (Thermo Scientific, MA, USA), purchased from Beyotime (#C3006). Briefly, the cells were incubated in medium containing the indicated concentrations of MA or/and CQ for 24 h. Confocal images was obtained using an Axio-Imager_LSM-800 confocal microscope (Zeiss, Jena, Germany) equipped with a 63× oil immersion objective. 

### 4.10. Lysosensor Assay

Lysosomal staining with the fluorescent acidotropic probe, LysoSensor™ Green DND-189, was conducted according to the manufacturer’s instructions (YEASEN, Shanghai, China). Briefly, cells were seeded in 6-well plates and treated with MA or/and CQ for 24 h, then harvested and incubated with Lysosensor™ Green DND-189 (1 µM ) for 1 min on ice followed by flow cytometric analysis. For detection of acidic vacuoles, cells were seeded on confocal dishes. After MA or/and CQ treatment for 24 h, the cells were incubated with 0.5 μM Lysosensor™ Green DND-189 for 2 h and subsequently analyzed by a confocal laser scanning microscope (Carl Zeiss, Jena, Germany).

### 4.11. sEVs Isolation and Purification

sEVs were isolated and purified from MDA-MB-231 and MCF-7 cell culture supernatant by differential ultracentrifugation as described previously [38]. Briefly, cells were cultured in 1640 and DMEM supplemented with 10% sEV-depleted fetal bovine serum for 48 h, then cell culture supernatant was harvested and centrifuged at 2000× *g* for 10 min to remove cells, 10,000× *g* for 30 min to remove cell debris. The resulting supernatant was filtered through 0.2 μm sterile filters (Millipore), followed by ultracentrifugation at 110,000× *g* for 70 min. The pellets were re-suspended in PBS and ultracentrifuged again at 110,000× *g* for 70 min. All centrifugations were performed at 4 °C. 

### 4.12. Nanoparticle Tracking Analysis (NTA) 

After purification of sEVs, the size distribution and concentration of the samples were measured using an NS300 NTA instrument (Malvern Instruments, Ltd., Malvern, UK). The data were analyzed using NTA software (NTA version 2.3 build 0017, Malvern Instruments Ltd.). To perform the measurement, sEVs samples were filtered using 0.22 μm filter membranes and diluted 10- to 100-fold in PBS to make the number of particles in the field of view about 100 per frame.

### 4.13. sEVs Quantification

To measure the quantity of sEVs in culture supernatants, AChE activity (EXOCET Exosome Quantification kit; System Biosciences) was determined according to the manufacturer’s protocol. Briefly, purified sEVs extracted from MDA-MB-231 cells untreated or treated with MA (4 μM) or/and CQ (20 μM) were incubated with color indicator dithibionitrobenzoic (DNTB, 0.1 mM) and substrate acetylthiocholine (Ach, 1.25 mM) in 96-well microtiter plates for 30 min, then the absorbance values were read at 412nm at 5-minute time intervals.

### 4.14. Statistical Analysis

Statistical analysis was conducted with GraphPad Prism software (La Jolla, CA, USA). Data were shown as means ± standard deviation (SD). One-way ANOVA or two-sample equal variance Student’s t-test method was used to determine statistical significance. *p* < 0.05 was considered statistically significant. 

## 5. Conclusions

Overall, we demonstrated that MA has an antiproliferative effect and inhibits breast cancer cell migration and invasion by preventing EMT. In addition, MA promotes autophagosome formation by inactivating the RIP1-mediated AKT/mTOR signaling pathway and restrains autophagosome degradation by reducing lysosomal acidity, thus inducing secretory autophagy in breast cancer cells. Therefore, MA may be considered as a potential autophagy inhibitor, secretory autophagy may be regarded as an alternative pathway of the autophagy-lysosome system, and autophagic sEVs might be used as biomarkers to monitor lysosome function.

## Figures and Tables

**Table 1:** IC50 for MA and paclitaxel (PTX) [22] in MCF7 and MDA-MB-231 cells.

Cell Lines	Drug	IC50 (μM)
MCF-7	MA	2.86 ± 0.19
	PTX	0.0157 ± 0.0065 [22]
MDA-MB-231	MA	7.87 ± 0.30
	PTX	0.0017 ± 0.0005 [22]

## Data Availability

The original data are available from the correspondent author on request.
